# TRIM72 Alleviates Muscle Inflammation in mdx Mice via Promoting Mitophagy-Mediated NLRP3 Inflammasome Inactivation

**DOI:** 10.1155/2023/8408574

**Published:** 2023-01-18

**Authors:** Mengli Wu, Huan Li, Juanjuan He, Jiahui Liang, Yanmei Liu, Weixi Zhang

**Affiliations:** Department of Neurology, The First Affiliated Hospital, Sun Yat-sen University, Guangdong Provincial Key Laboratory of Diagnosis and Treatment of Major Neurological Diseases, National Key Clinical Department and Key Discipline of Neurology, Guangzhou 510080, China

## Abstract

Chronic muscle inflammation exacerbates the pathogenesis of Duchenne muscular dystrophy (DMD), which is characterized by progressive muscle degeneration and weakness. NLRP3 (nucleotide-binding domain and leucine-rich repeat pyrin domain containing 3) inflammasome plays a key role in the inflammatory process, and its abnormal activation leads to a variety of inflammatory or immune diseases. TRIM72 (MG53) is a protective myokine for tissue repair and regeneration. However, little is known about the potential impact of TRIM72 in the crosstalk between mitophagy and inflammatory process of DMD. Here, 10-week-old male mdx mice were injected intramuscularly with adeno-associated virus (AAV-TRIM72) to overexpress TRIM72 protein for 6 weeks. Then, skeletal muscle samples were collected, and relevant parameters were measured by histopathological analysis and molecular biology techniques. C2C12 cell line was transfected with lentivirus (LV-TRIM72) to overexpress or siRNA (si-TRIM72) to suppress the TRIM72 expression for the following experiment. Our data firstly showed that the TRIM72 expression was decreased in skeletal muscles of mdx mice. Then, we observed the increased NLRP3 inflammasome and impaired mitophagy in mdx mice compared with wild type mice. In mdx mice, administration of AAV-TRIM72 alleviated the accumulation of NLRP3 inflammasome and the consequent IL-18 and IL1*β* maturation by inducing autophagy, while this protective effect was reversed by chloroquine. Mitochondrial reactive oxygen species (mtROS), as a recognized activator for NLRP3 inflammasome, was attenuated by TRIM72 through the induction of mitophagy in C2C12 cells. Additionally, we proposed that the TRIM72 overexpression might promote mitophagy through both the early stage by PI3K-AKT pathway and the late stage by autolysosome fusion. In conclusion, the current study suggests that TRIM72 prevents DMD inflammation via decreasing NLRP3 inflammasomes and enhancing mitophagy. Collectively, our study provides insight into TRIM72 as a promising target for therapeutic intervention for DMD.

## 1. Introduction

Duchenne muscular dystrophy (DMD) is a serious and progressive inherited human myopathy derived from mutations in X-linked recessive gene encoding dystrophin, a membrane protein that maintains structural stability and integrity of myofiber [1]. Deficiency of dystrophin could alter the permeability of muscle cells, leading to influx of massive Ca^2+^ and rupture of mitochondria, causing increased reactive oxygen radicals [2]. This persistent damage of myofibers eventually results in infiltration of immune cells and chronic inflammation, which in turn inhibits the muscle regeneration and leads to muscle fibrosis, necrosis, and death [3]. DMD patients have to endure sustained muscle inflammation, aggravated respiratory and/or heart failure, and finally completed paralysis to death [4].

NLRP3 (nucleotide-binding domain and leucine-rich repeat pyrin domain containing 3) inflammasome is an important part of inflammation, and a complex consists of NLRP3, apoptosis-associated speck-like protein (ASC), and procaspase-1. Activation of NLRP3 inflammasome would facilitate procaspase-1 self-cleavage into cleaved-caspase-1, as well as transforming IL-1*β* and IL-18 from precursor to active forms [5]. However, excessive inflammatory cytokines will cause hyperinflammation and detrimental immune response if without appropriate regulation [6]. Though NLRP3 inflammasome primarily presents in immune and inflammatory cells, emerging evidences have indicated that NLRP3 also appears within muscle cells of DMD and other myopathies [7, 8].

Mitophagy, a self-protective process critical for scavenging impaired mitochondria, could negatively regulate NLRP3 inflammasome [9, 10]. Dysfunctional mitochondria could release mitochondrial DNA (mtDNA) and mitochondrial reactive oxygen species (mtROS), which are required for NLRP3 activation [11]. Skeletal muscles of mdx mice present with abnormal mitochondrial structure and impaired mitophagy prior to onset of myofiber necrosis [12]. Restoring mitophagy ameliorates muscle function and pathophysiology of mdx mice [2, 13]. Therefore, modulating mitophagy might be a feasible way to decrease NLRP3 and treat DMD.

A tripartite motif family protein (TRIM72), also known as MG53, is mainly generated in the skeletal muscle and heart [14]. Recently, researches point out that the TRIM72 overexpression could facilitate mitophagy in atrophic skeletal muscle of chronic kidney disease model and C2C12 cells [15]. Autoantibodies aiming at TRIM72 exacerbate idiopathic inflammatory myopathy [16]. Regulation of TRIM72 might mitigate inflammation through NF-*κ*B pathway both in vivo and vitro [17–20]. Nevertheless, whether TRIM72 could influence the inflammation of mdx mice and how TRIM72 works in the crosstalk between inflammation and mitophagy in mdx mice remains unclear. Therefore, in this study, we aimed to uncover the effects of TRIM72 on mdx mice and clarify the underlying mechanisms.

## 2. Materials and Methods

### 2.1. Mice Handing and Adeno-Associated Virus (AAV) Administration

Animal protocols used in this study have been reviewed and approved by the Institutional Animal Care and Use Committee (IACUC), Sun Yat-sen University. Animals were randomly selected and assigned to different treatment groups, and efforts were made to minimize the animal numbers and their suffering. All mice (about 10 weeks) were housed on a 12/12 hr light/dark cycle under temperature-controlled room (24°C) with food and water ad libitum. Intraperitoneal injection of sodium pentobarbital (50 mg/kg) was applied to anesthetize the mice. The male mdx mice (C57BL/10ScSn-Dmd^em3CD4^/Gpt) and relative wild type (WT) background mice (C57BL/10) were purchased from the GemPharmatech Company (Jiangsu, China).

AAV with empty vector (AAV-EV) and mouse TRIM72 vector (AAV9-TRIM72-3xflag, AAV-TRIM72) were purchased from Hanbio Biotechnology Company (Shanghai, China). 10-week-old male mdx mice were randomly divided into four groups: blank control mdx mice, AAV-EV-treated negative control mdx mice, AAV-TRIM72-treated mdx mice, and AAV-TRIM72 plus chloroquine (CQ, C6628, Sigma-Aldrich, Germany)-treated mdx mice. In AAV-treated mdx mice, each tibial anterior muscle received 25 *μ*l (1.5^∗^10^12^ vg/mL, liquid formulation) injection by intramuscular injection. After 6 weeks of injection, mice were euthanized, and tibial anterior muscles were collected after above interventions. Intraperitoneal injection of CQ (80 mg/kg/day) was administered for 4 days before execution.

### 2.2. Tissue Collection and HE Staining

Tibialis anterior muscles were removed from mice and frozen rapidly with precooled isopentane (78-78-4, ThermoFisher, America) in liquid nitrogen and preserved at −80°C. The frozen cross-sections of muscle were achieved under standard cryostat with clean and sharp blade and sectioned at a thickness of 10 *μ*m. Some of unfixed slices were stained with MitoTracker Red CMXRos (M7512, ThermoFisher, America) to label mitochondria. Remaining muscle slices were fixed with precooled acetone (A949-4, Sigma-Aldrich, Germany) for 10 mins and stored in -80°C for following experiment.

Protocol of hematoxylin and eosin (HE) staining kit (G1120, Solarbio, Beijing, China) was as follows: (a) restore fresh frozen sections to room temperature, (b) stain nucleus with Hematoxylin Solution for 5 min and rinse in running tap water, (c) differentiate with Differentiation Solution for 3 min and wash with tap water twice for 2 min each, (d) redyeing with Eosin Y Aqueous Solution for 2 min and quickly wash in deionized water, (e) dehydrate in alcohol (75%, 85%, 95%, and 100% alcohol (I)), each for 2~3 s, and rinse in 100% alcohol (II) for 1 min, and (f) transparent by xylene and seal with resinene.

### 2.3. Western Blot Assay

Briefly speaking, the muscle tissue or cellular proteins were lysed using radio immunoprecipitation assay (RIPA, 89900, ThermoFisher, America) lysis buffer with 1% phenylmethanesulfonyl fluoride (PMSF, ST505, Beyotime, Shanghai, China) and 1% phosphatase inhibitors (78426, ThermoFisher, America). Proteins were separated on SDS-PAGE gels (Epizyme, Shanghai, China) via electrophoresis and then transferred to polyvinylidene difluoride membrane (PVDF, IPVH00010, Millipore, America) and blocked with 5% bovine serum albumin (BSA, B2064, Sigma-Aldrich, Germany). Membranes were then incubated with the primary antibodies overnight (4°C) and washed with TBST. Secondary antibodies were added for 1 h at 37°C. Finally, we visualized bands with enhanced chemiluminescence kit (FDbio Science, FD8020, Hangzhou, China) and measured the densitometry with ImageJ software. All the primary antibodies information were collected and shown in Supplemental Table 1.

### 2.4. Immunofluorescence and MitoTracker Labelling

For both muscular and cellular slices, they were rinsed with phosphate-buffered saline (PBS) for three times. Cells were then fixed with 4% paraformaldehyde for 20 min. Next, 0.3% Triton X-100 (T8787, Sigma-Aldrich, Germany) was subjected to permeabilization for 20 min and 5% BSA blocking for 1 h. Next, slides were incubated with primary antibody at 4°C overnight. After PBS washing for three times, the corresponding secondary antibody was incubated for 2 hours at room temperature and sealed with sealant containing DAPI (F6057, Sigma-Aldrich, Germany) for 5 min. The final results were visualized via immunofluorescence microscope at 550 nm for MitoTracker Red and corresponding wavelength for fluorescent secondary antibodies.

For slices that require mitochondria labelling, they were prestained via MitoTracker Red CMXRos at 500 nM for 30 min at room temperature. The results were assessed by ImageJ software.

### 2.5. Enzyme-Linked Immunosorbent Assay

The amount of inflammatory cytokine IL1*β* from mice serum was measured by commercial ELISA kit (KE10003, Proteintech, Wuhan, China) according to the manufacturer's instructions.

### 2.6. mtROS Generation and Detection

Antimycin A (AA, A8674, Sigma-Aldrich, Germany), an inhibitor of mitochondrial respiratory membrane complexes I and III, was used at a concentration of 10 *μ*M for 24 h to increase the mtROS levels. MitoSOX™ Red Mitochondrial Superoxide Indicator (M36008, ThermoFisher, America) was utilized to detect the activity of mtROS. According to the manufacturer's instructions, we administered MitoSOX dye to cells with a final concentration of 5 *μ*M for 30 min at 37°C. After washing with PBS for 3 times, cells were sealed with antifluorescence quenching sealant including DAPI, and then, images were captured under fluorescence microscopy.

### 2.7. Quantitative Real-Time Polymerase Chain Reaction (qRT-PCR)

According to the manufacturer's instructions, total RNA was extracted from samples by RNAzol reagent (RN190, MRC, China). PrimeScript RT reagent kit (RR047A, Takara, Japan) was used to reverse-transcribed RNA to cDNA, and qRT-PCR was performed using CYBR Green dye. After normalization to GAPDH mRNA using the comparative cycle threshold (Ct) method, we analyzed the relative quantification of mRNA levels—mouse GAPDH primers: forward, 5′-AGGTCGGTGTGAACGGATTTG-3′; reverse, 5′-TGTAGACCATGTAGTTGAGGTCA-3′. Mouse TRIM72 primers: forward, 5′-ACTGAGCATCTACTGCGAGC-3′; reverse, 5′-ACGATGACCACGGTGAGAAC-3′.

### 2.8. In Vitro Cell Culture and Treatment

C2C12 is an immortalized mouse myoblast cell line which is widely used in muscle models in vitro. They were adherent and originally obtained by Yaffe and Saxel at the Weizmann Institute of Science in Israel in 1977 [21]. C2C12 cells were cultured in DMEM (C11995500BT, Gibco, America) supplemented with 10% fetal bovine serum (FBS, 10099-141C, Gibco, America). They were incubated in a 5% CO_2_ environment at 37°C. To trigger mitophagy, cells were incubated with 50 *μ*M carbonyl cyanide m-chlorophenylhydrazone (CCCP, C6700, Solarbio, Beijing, China) for 12 h. CQ at concentration of 10 *μ*M for 72 h was used to inhibit autophagic flux.

### 2.9. Lentivirus (LV) and Small Interfering RNA (siRNA) Transfection

LV with empty vector (LV-EV) or mouse TRIM72 vetor (LV-TRIM72-3xflag-ZsGreen-PURO, LV-TRIM72) obtained from Hanbio Biotechnology Company was transfected into C2C12 cell line to establish a negative control group or TRIM72 overexpression group. LV with polybrene (10 *μ*g/mL, HB-PB, Hanbio, Shanghai, China) and C2C12 cells was cocultured for 24 h. Next, we replace the viral media with fresh media and recover the cells for 48 h. Cells were then selected with puromycin (1.0 *μ*g/mL, ST551, Beyotime, Shanghai, China) to construct a stable strain and monitored under immunofluorescence microscopy. Western blot and qRT-PCR were also applied to verify the protein expression.

Control siRNA (si-control) and siRNA targeting mouse TRIM72 (si-TRIM72) were provided by Qingke Company. One day after seeding, cells were transiently transfected with 50 nM siRNA at 50% confluence combining with the Lipofectamine 3000 (L3000015, Invitrogen, America). The knockdown efficiency of the interested protein was measured with western blot. The sequence of si-TRIM72 was as follows: forward, 5′-CGGCAAGGCUAGAUAUCCATT-3′ and reverse, 5′-UGGAUAUCUAGCCUUGCCGTT-3′.

### 2.10. Statistical Analysis

All statistical analyses were performed with GraphPad Prism software. Data are presented as the mean ± standard error of the mean. The comparisons between two datasets were analyzed by two-tailed unpaired student *t*-test, and one-way ANOVA analysis of variance with Tukey's post hoc test was used for comparisons of multiple groups. For all tests, values of *p* < 0.05 were considered to indicate a statistically significant difference. Specifics regarding results and the statistical tests performed are provided in each figure legend.

## 3. Results and Discussion

### 3.1. TRIM72 Overexpression and Knockdown In Vivo and Vitro

TRIM72, which remarkably expressed in skeletal and heart muscles, is a protein implicated in membrane repairment [22, 23]. Considering that DMD disease manifests as membrane impairment, we explored how TRIM72 expressed in mdx mice. The endogenous TRIM72 expression in tibial anterior muscles of mdx mice showed a significantly reduction. Immunofluorescent imaging showed disordered and broken TRIM72 localization in the tibial anterior slice of mdx mice (Supplemental Figure 1(a)). Correspondingly, western blot verified a less expression in mdx mice again (Supplemental Figures 1(b) and 1(c)). Consistent with previous studies, those indicated an indispensable impact of TRIM72 on repairment of DMD [24].

AAV vectors have been recognized as a leading tool for therapy due to the low toxicity and effectiveness of gene delivery [25, 26]. AAV9 is one of the most commonly used serotypes targeting skeletal and cardiac muscle [27, 28]. Transferring normal dystrophin gene into dystrophic muscles of DMD patients seems to be a logically feasible therapy [29]. However, the large size of this gene increased the difficulty of virus packaging and restricted its application [30]. Herein, we injected AAV-TRIM72 (designed with 3xflag tag) intramuscularly through the tibial anterior muscle to overexpress TRIM72 and explore its function in vivo.

**Figure 1:**
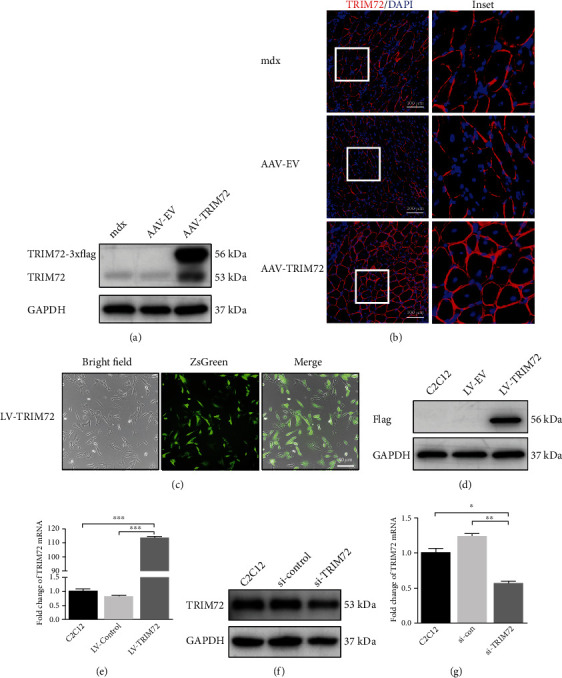
TRIM72 overexpression and knockdown in vivo and vitro. (a) Western blot analysis of tibial anterior muscle extracts using antibodies against TRIM72 after AAV-EV or AAV-TRIM72 transfection in mdx mice. AAV-TRIM72 was designed with 3xflag tag. (b) Representative immunofluorescence images of TRIM72 in tibial anterior muscle of mdx mice transfected with or without AAV. Scale bar, 100 *μ*m. (c) Fluorescence indicated the successful transfection of LV-TRIM72 in C2C12 cells. Scale bar, 50 *μ*m. (d) Western blot analysis of C2C12 cell extracts using antibodies against Flag after lentivirus transfection. LV-TRIM72 was designed with 3xflag tag. (e) The expression of TRIM72 mRNA in C2C12 cells with or without lentivirus transfection for 72 h. (f) C2C12 cells were transfected with si-TRIM72 for 72 h, and western blot was performed with antibodies against TRIM72. (g) The expression of TRIM72 mRNA in C2C12 cells with or without siRNA transfection for 72 h. Data were expressed as mean ± SEM. ^∗^*p* < 0.05, ^∗∗^*p* < 0.01, and ^∗∗∗^*p* < 0.001. AAV-EV: adeno-associated virus with empty vector; LV-EV: lentivirus with empty vector.

At age of 10 weeks, mdx mice were treated with AAV-TRIM72 or AAV-EV. After 6 weeks, we collected the tibial anterior muscles in the three groups including control mdx mice, AAV-EV mdx mice, and AAV-TRIM72 mdx mice. TRIM72 protein was significantly elevated in AAV-TRIM72 mice (Figure 1(a)). TRIM72-3xflag and TRIM72 represented exogenous and endogenous expressions, respectively. Immunofluorescent imaging indicated that disordered and broken TRIM72 localization had been obviously restored in AAV-TRIM72 mdx mice (Figure 1(b)). Therefore, our results indicated that the AAV-TRIM72 model was successfully established, and TRIM72 magically repaired the membrane of mdx mice.

Moreover, we applied lentivirus and siRNA to overexpress or knockdown TRIM72 in C2C12 cells. The green fluorescence indicated the successful transfection of LV-TRIM72 (LV-TRIM72-3xflag-ZsGreen-PURO) into C2C12 cells (Figure 1(c)). Western blot using anti-Flag antibody verified the notable expression once again (Figure 1(d)). LV-TRIM72 cells showed a significantly increased mRNA (Figure 1(e)). Furthermore, si-TRIM72 transfection in C2C12 cells showed an efficient knockdown fact by western blot and qRT-PCR (Figures 1(f) and 1(g)).

### 3.2. TRIM72 Decreased NLRP3 Inflammasomes by Inducing Mitophagy in mdx Mice

Consistent with previous study, our results showed a distinction of inflammatory response between WT and mdx mice [7, 31]. HE indicated that mdx mice exhibited significant histopathology with abnormal fibers, centrally located nuclei, and inflammatory infiltration (Supplemental Figure 2(a)). Immunofluorescence images also showed that NLRP3 was even positioned inside myofibers and significantly upregulated in muscle tissues of mdx mice (Supplemental Figure 2(b)). In a similar form, the protein expression of NLRP3 inflammasome containing NLRP3, ASC, and caspase-1 and proinflammatory cytokines of the interleukin family, such as IL18 and IL-1*β*, were increased in mdx mice (Supplemental Figures 2(c) and 2(d)). Moreover, IL-1*β* content in the serum of mdx mice was also significantly augmented by ELISA assay (Supplemental Figure 2(e)).

**Figure 2:**
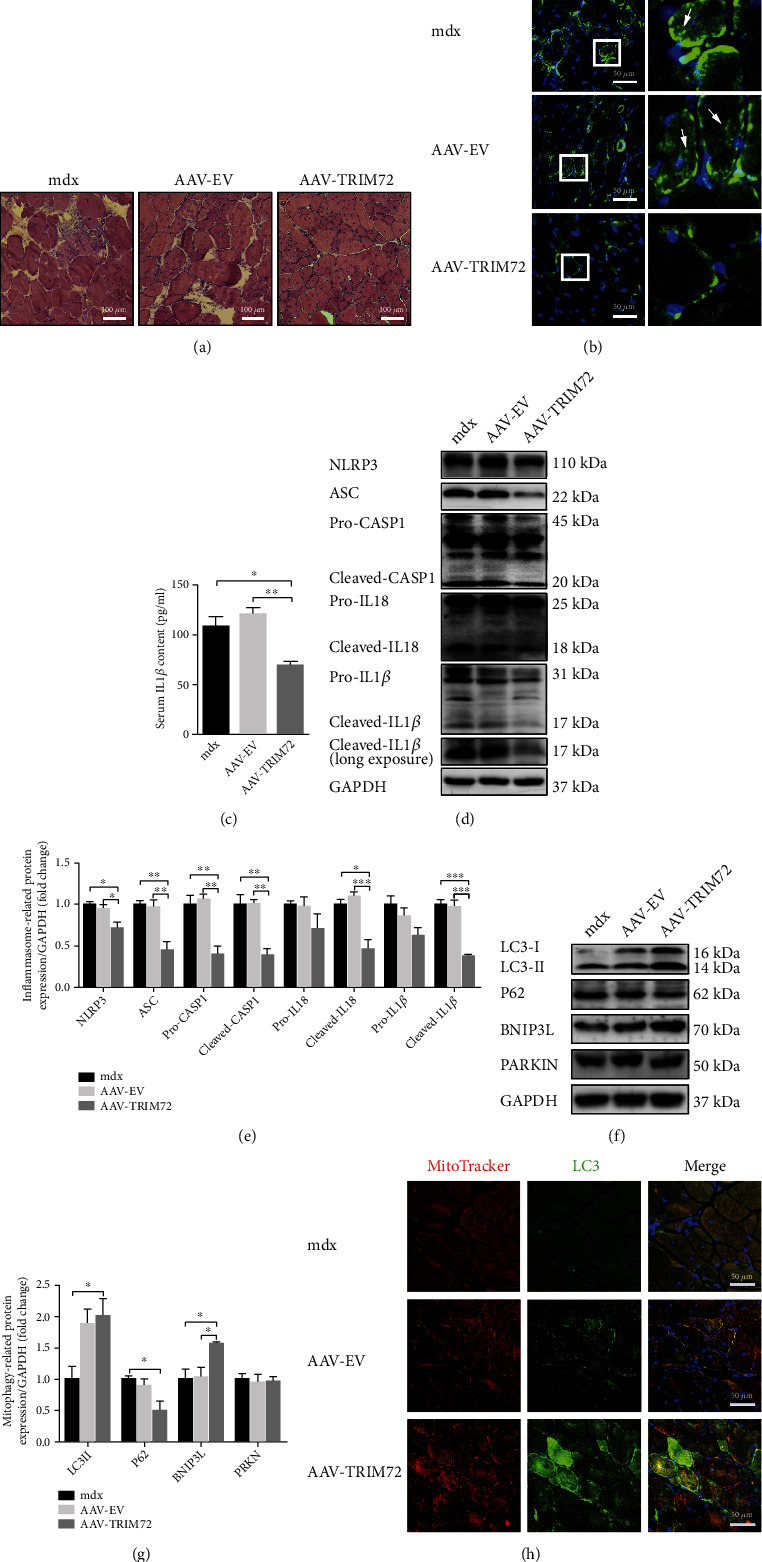
Decreased NLRP3 inflammasomes and increased mitophagy in AAV-TRIM72 *mdx* mice. (a) Representative HE staining images of tibial anterior muscles in mdx mice with or without AAV transfection. Scale bar, 100 *μ*m. (b) Immunofluorescence was performed with specific antibody targeting NLRP3. As shown by *arrows* in the magnification *inset*, NLRP3 dots were reduced in AAV-TRIM72 mdx mice. Scale bar, 50 *μ*m. (c) Serum IL-1*β* level detected by ELISA assay, *n* = 4. (d, e) Western blot analysis and quantification of NLRP3, ASC, IL18, caspase-1, and IL-1*β* in three mdx groups, *n* = 4. (f, g) Immunoblot analysis and quantification of P62, LC3, BNIP3, and PARKIN in lysates of three mdx groups, *n* = 4. (h) Representative immunofluorescence images of LC3, MitoTracker, and the merged images in three mdx groups. Scale bar, 50 *μ*m. Data were expressed as mean ± SEM. ^∗^*p* < 0.05, ^∗∗^*p* < 0.01.

HE staining revealed that inflammatory response was suppressed in AAV-TRIM72 mdx mice than untreated and AAV-EV mdx mice (Figure 2(a)). Of note, NLRP3 positive dots decreased in the skeletal cells of AAV-TRIM72 mdx mice, indicating that TRIM72 could reduce the activation of NLRP3 (Figure 2(b)). Likewise, western blot also witnessed a reduced protein levels of NLRP3 after the TRIM72 overexpression, as well as a same tendency in consequent protein ASC, caspase-1, IL-18, and IL-1*β* (Figures 2(d) and 2(e)). Furthermore, ELISA analysis also showed that serum content of IL-1*β* was significantly downregulated after AAV-TRIM72 treatment (Figure 2(c)). Together, findings above revealed that TRIM72 could alleviate NLRP3 inflammasome activation and inflammatory cytokine secretion in mdx mice.

Impaired autophagic flux has been recognized in the muscle of mdx mice [32]. LC3 is a molecular which could tether to the lipid bilayer of autophagosome, extend the isolated membrane by gathering lipids, and facilitate the self-fusion and formation of autophagosome [33]. Generally, low ratio of LC3-II:LC3-I suggests insufficient autophagosome formation, while high LC3-II:LC3-I reflects inadequate degradation of autophagosome or increased autophagosome formation [2]. P62 is enhanced when autophagy is inhibited. It is interesting that our data showed a higher LC3-II:LC3-I and P62 in mdx mice, which was consistent with previous studies but lack of explanation (Supplemental Figures 2(f) and 2(g)) [2, 34]. Some researchers have pointed that though LC3 is an indispensable structure of autophagosome and a typically monitored autophagy-related protein, the preinitiation and degradation processes of autophagy do not rely on it [33, 35]. Therefore, we proposed that elevated LC3-II reflected the body's spontaneous and protective autophagic response when facing inflammatory, while increased P62 and decreased ATG5 or LAMP1 in mdx mice reflected insufficient and weak autophagic flux. Mitophagy-related protein, such as BNIP3L, was significantly reduced, while PARKIN was slightly reduced in the muscle of mdx mice (Supplemental Figures 2(f) and 2(g)). Together, our results indicated that mitophagy was insufficient in the muscle of mdx mice.

Then, we attempted to uncover the regulatory mechanism whereby TRIM72 prevents the inflammation aggravation. Results showed a LC3-II increase and P62 degradation in AAV-TRIM72 mdx mice. TRIM72 significantly elevated the BNIP3L protein expression, but with little variation in PARKIN level, indicating that TRIM72 might activate mitophagy through receptor-mediated not PINK-PARKIN pathway (Figures 2(f) and 2(g)). Additionally, we used MitoTracker and LC3 to label mitochondria and autophagosome, respectively. As shown in immunofluorescence, TRIM72 increased LC3 dots and facilitated the combination of mitochondria and autophagosome in mdx mice (Figure 2(h)).

### 3.3. Inhibition of Autophagy Abrogated the Inhibitory Effects of TRIM72 on NLRP3 Activation in mdx Mice

**Figure 3:**
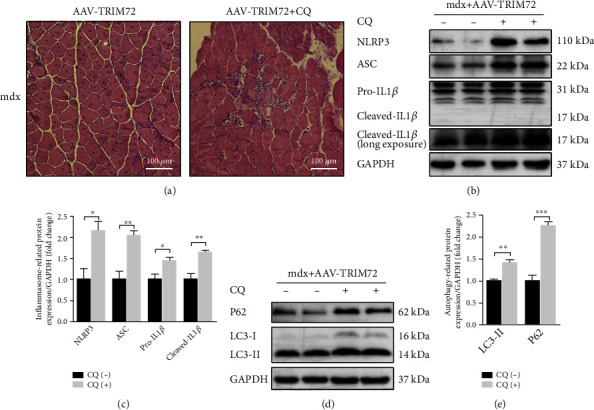
Inhibition of autophagy abrogated the inhibitory effects of TRIM72 on NLRP3 activation. (a) Representative histology images and inflammatory response reflected by HE staining in tibial anterior muscles of AAV-TRIM72 mdx mice with or without CQ treatment. Scale bar, 100 *μ*m. (b, c) Western blot and quantification of inflammasome-related proteins NLRP3, ASC, and IL1*β* expression in tibial anterior muscles of AAV-TRIM72 mdx mice with or without CQ treatment, *n* = 4. (d, e) Western blot and quantification of autophagy-related proteins P62 and LC3 expression in AAV-TRIM72 mdx mice with or without CQ treatment, *n* = 4. Data were expressed as mean ± SEM. ^∗^*p* < 0.05, ^∗∗^*p* < 0.01, and ^∗∗∗^*p* < 0.001. CQ: chloroquine.

CQ could impair autophagy flux in phase of autolysosome fusion [36]. HE staining indicated that blockade of autophagy reversed the anti-inflammatory effect of AAV-TRIM72 (Figure 3(a)). Administration of CQ not only hindered the exhaustion of autophagosomes presenting with elevated LC3-II transformation and P62 but also NLRP3 inflammasome expression and IL-1*β* release (Figures 3(b)–3(e)). Data above suggested that autophagy was indispensable for anti-inflammatory function of TRIM72.

### 3.4. TRIM72 Could Scavenge mtROS through Mitophagy Induction in C2C12 Cells

**Figure 4:**
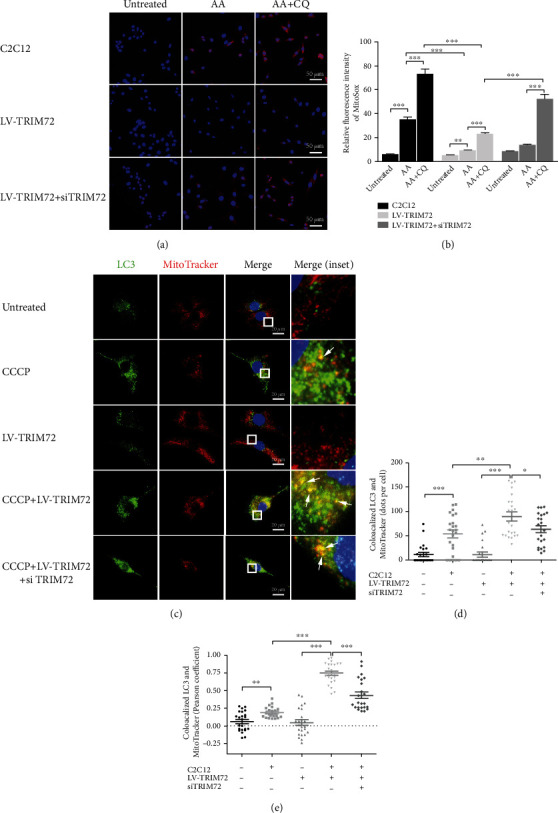
TRIM72 could scavenge mtROS through mitophagy induction in C2C12 cells. (a, b) Representative images and statistical data of MitoSOX Red fluorescence in C2C12 cells, LV-TRIM72 cells, and LV-TRIM72 + si-TRIM72 cells. Cells were cocultured with or without antimycin A (AA, 10 *μ*M) and/or chloroquine (CQ, 50 *μ*M) for 24 h. Scale bar, 50 *μ*m. Data were obtained from 15 randomly selected cells from 3 independent experiments. (c) Representative immunofluorescence images of LC3, MitoTracker, and the merged images in C2C12 cells. Cells were cultured with or without CCCP (50 *μ*M), LV-TRM72, or si-TRIM72 for 12 h. Scale bar, 20 *μ*m. (d, e) Statistical data of colocalized LC3 and MitoTracker was analyzed by Pearson's coefficient or dots per cell. Data were obtained from 24 randomly selected cells from 3 independent experiments. Data were expressed as mean ± SEM. ^∗^*p* < 0.05, ^∗∗^*p* < 0.01, and ^∗∗∗^*p* < 0.001. AA: antimycin A; CCCP: carbonyl cyanide m-chlorophenylhydrazone.

The generation of mtROS by damaged mitochondria plays a crucial role in mdx muscle progression [2]. Antimycin A (AA), which could inhibit the electron transport chain of mitochondria and depolarize mitochondria, was widely used to induce mtROS [37]. LV-TRIM72 suppressed the mtROS generation which was significantly elevated in AA treated cells, while this effect was partly reversed by si-TRIM72. As inhibitor of autophagy, CQ aggravated the fluorescence intensity of MitoSOX of AA-treated cells (Figures 4(a) and 4(b)). Together, healthy autophagy flux was required for mtROS elimination and antioxidative effect of TRIM72.

We further examined whether TRIM72 could influence mitophagy in C2C12 cells. CCCP, which was widely demonstrated to uncouple mitochondrial oxidative phosphorylation and trigger mitophagy, was applied to estimate mitophagy flux over here. Colocalized LC3/MitoTracker dots were increased in CCCP+LV-TRIM72 cells compared with only CCCP-treated cells. To confirm the result, si-TRIM72 was added to CCCP+LV-TRIM72 cells, and data showed a downregulated colocalization of LC3/MitoTracker. Contrary to CCCP-processed cells, LV-TRIM72 cells did not present a statistical significance compared with untreated C2C12 cells (Figures 4(c)–4(e)). Collectively, TRIM72 could promote the clearance of damaged mitochondria but have little effect on healthy mitochondria.

### 3.5. TRIM72 Facilitated the Late Stage of Mitophagy Flux

**Figure 5:**
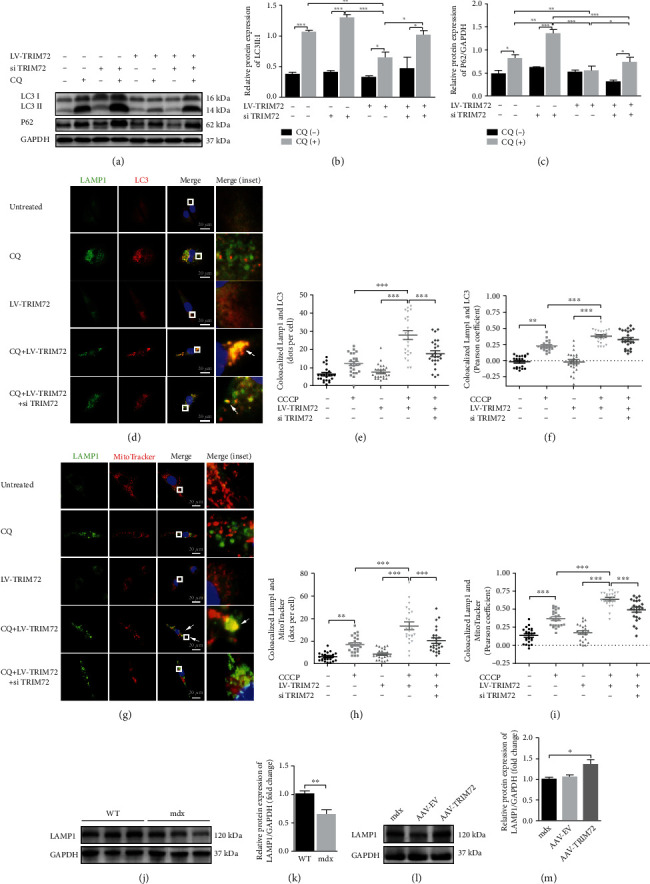
TRIM72 facilitated the late stage of mitophagy flux. (a–c) Western blot and statistical analysis of P62 and LC3 in C2C12 cells. Cells were cultured with or without CQ (10 *μ*M), LV-TRM72, or si-TRIM72 for 72 h, *n* = 4. (d) Representative immunofluorescence images of LAMP1, LC3, and the merged images in C2C12 cells. Cells were cultured with or without CQ (50 *μ*M), LV-TRM72, or si-TRIM72 for 24 h. Scale bar, 20 *μ*m. (e, f) Statistical data of colocalized LAMP1 and LC3 was analyzed by Pearson's coefficient or dots per cell. (g) Representative immunofluorescence images of LAMP1, MitoTracker, and the merged images in C2C12 cells. Cells were cultured with or without CQ (50 *μ*M), LV-TRM72, or si-TRIM72 for 24 h. Scale bar, 20 *μ*m. (h, i) Statistical data of colocalized LAMP1 and MitoTracker was analyzed by Pearson's coefficient or dots per cell. (j, k) Western blot and quantification of LAMP1 in tibial anterior muscle of WT and mdx mice, *n* = 6. (l, m) Western blot and quantification of LAMP1 in tibial anterior muscle of blank, AAV-EV and AAV-TRIM72 treated mdx mice, *n* = 4. Scale bar, 50 *μ*m. In (e, f) and (h, i), data were obtained from 24 randomly selected cells from 3 independent experiments. Data were expressed as mean ± SEM. ^∗^*p* < 0.05, ^∗∗^*p* < 0.01, and ^∗∗∗^*p* < 0.001.

Autolysosome formation means the completion of late stage in mitophagy [10]. Western blot showed that LV-TRIM72 decreased CQ-triggered LC3-II and P62 accumulation, while the result was reversed by si-TRIM72 (Figures 5(a)–5(c)). LAMP1, one of the capital protein components of the lysosomal membrane, coupled with LC3 by double-immunofluorescence staining was utilized for analyzing the fusion of lysosome and autophagosome [38]. Yellow dots suggested the colocalization of two targeted indicators. We observed the increasing dots but low colocalization of LC3/LAMP1 in CQ-treated cells compared with untreated cells. In particular, CQ + LV-TRIM72 reversed the decreased colocalization in CQ-treated cells. Addition of si-TRIM72 to CQ + LV-TRIM72 cells decreased the enhanced colocalization (Figures 5(d)–5(f)). Since mitochondria could be engulfed by autophagosome, the same function of TRIM72 was observed in the combination of mitochondria and lysosome (labeled by MitoTracker and LAMP1, respectively) (Figures 5(g)–5(i)). When cells were not administrated with CQ, we did not find significant difference after LV-TRIM72 treatment both in western blot and immunofluorescence (Figures 5(a)–5(i)). AAV-TRIM72 could also increase the LAMP1 expression which was deficient in mdx mice (Figures 5(j)–5(m)). Together, TRIM72 could restore the defective mitophagy flux at late stage.

### 3.6. TRIM72 Might Regulate the Early Stage of Mitophagy through PI3K-AKT Pathway

**Figure 6:**
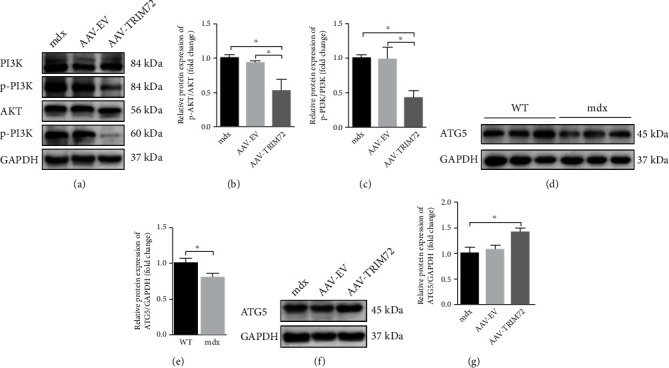
TRIM72 might regulate the early stage of mitophagy through PI3K-AKT pathway. (a–c) Protein levels of PI3K, AKT, and their phosphorylated forms in tibial anterior muscles of blank, AAV-EV, and AAV-TRIM72-treated mdx mice, *n* = 4. (d, e) Western blot and quantification of ATG5 in tibial anterior muscles of WT and mdx mice, *n* = 6. (f, g) Western blot and quantification of ATG5 in tibial anterior muscles of three mdx groups, *n* = 4. Data were expressed as mean ± SEM. ^∗^*p* < 0.05.

PI3K-AKT-mTOR pathway is essential for autophagosome initiation at the early phase [39]. Here, our results demonstrated that AAV-TRIM72 decreased the p-AKT and p-PI3K level in mdx mice (Figures 5(c)–6(a)). Furthermore, we assessed the ATG5 expression, a critical protein for phagophore elongation. As expected, AAV-TRIM72 restored the decreased ATG5 in mdx mice (Figures 5(g)–6(d)). Over all, TRIM72 might regulate the early stage of mitophagy.

## 4. Discussion

For decades, incremental efforts have been made to explore the therapy for DMD. However, it is hard for the current treatment to stop the ruthless muscle tissue and function loss of this disease, which ultimately leads to premature death. TRIM72, a hot unearthed protein in recent years, exhibits a protective and positive effect both in vivo and vitro [40]. Accumulating researches lighted that TRIM72 plays a vital role in muscle regeneration and might be a promising biomarker and therapy for DMD [41]. Nevertheless, limited studies have investigated the role of TRIM72 on muscle protection through the regulation between autophagy and inflammation. Here, our study demonstrates that TRIM72 might inactivate and scavenge NLRP3 inflammasomes by enhancing mitophagy, thereby alleviating mdx inflammation.

Activation and accumulation of NLRP3 inflammasomes have been involved in associated with pathogenesis of many inflammatory diseases [5]. Previous study suggested that downregulation of the NLRP3 inflammasome could improve muscle function of DMD [7, 31, 42]. Impaired membrane integrity caused by dystrophin deficiency could increase the influx of Ca^2+^, leading to mitochondria dysfunction and ROS release [2]. Inhibiting Ca^2+^ flux might impede the assemble and activation of NLRP3 inflammasome in macrophages [43]. TRIM72, a key role in cell membrane repairment and tissue regeneration, is a myokine which has protective effects on multiple tissues including muscle, heart, lungs, kidneys, and brain [44]. It is interesting that partial TRIM72 could be secreted into blood and dispersed throughout the body participating in membrane repairment and other physiological process [40, 45–47]. This might explain the decreased serum IL1*β* secretion in AAV-TRIM72 mdx mice (Figure 2(c)). Moreover, recent researches revealed an additional anti-inflammatory role of TRIM72 concerning chronic tissue injury, sepsis, or viral infection [20, 48–50]. TRIM72 might suppress inflammation through regulating ryanodine receptor-mediated Ca^2+^ signal or PPAR *α* [19, 50]. There was also study pointed out that TRIM72 suppressed NLRP3 inflammasomes in LPS-induced neuroinflammation [51]. Targeting the anti-inflammatory role of TRIM72 might be a promising application for mdx pathology alleviation. Consistent with previous studies, we found a prominent accumulation of NLRP3 inflammasome and inflammatory cytokine in mdx mice (Supplemental Figures 2(a)-2(e)). There was also a noticeable reduction of TRIM72 protein in mdx mice (Supplemental Figure 1). Particularly, the overexpression of TRIM72 decreased NLRP3 inflammasomes and inhibited the following maturation of caspase-1, IL-1*β*, and IL18, eventually alleviating inflammatory response of mdx mice (Figures 2(a)–2(e)). Thus, our results suggested a protective and anti-inflammatory role of TRIM72 in DMD.

Autophagy process is necessary for normal immune response when encountering inflammatory factors [33, 52]. Appropriate autophagy could remove useless organelles and inflammasomes and thus protect the host. Mitophagy, one of the most common types of autophagy, is an intracellular process delivering damaged mitochondria to lysosomes for digestion [53]. The interrelationship between NLRP3 inflammasome and autophagy has been demonstrated by numerous researches during the past decades. It is not a simple upstream-downstream relationship but a bidirectional causality indispensable for the balance between normal anti-inflammatory response of host and aberrant hyperinflammation. NLRP3 inflammasome could induce mitophagy and mitophagy can suppress inflammation. Therefore, insufficient induction of mitophagy might aggravate inflammatory response and result in cell apoptosis and necrosis. Excessive inflammation could destruct the cell and tissue, which in turn stimulate mitophagy enhancement to remove NLRP3 inflammasome components and corresponding activators [54]. Mitochondria dysfunction which existed before muscle breakdown in mdx mice indicated that impaired mitochondria might be a contributor to muscle pathology instead of a consequence [12]. Our study also verified the impairment of mitophagy-related indicators in mdx mice (Supplemental Figures 2(f) and 2(g)). Most importantly, the overexpression of TRIM72 enhanced mitophagy (Figures 2(f)–2(h)). Blockade of AAV-TRIM72-induced autophagy by CQ reversed the positive effect and leaded to increased NLRP3 and hypersecretion of IL-1*β* (Figure 3). Overall, we demonstrated that TRIM72 inhibited NLRP3 inflammasomes by enhancing autophagy in mdx mice.

Impaired mitophagy in DMD results in the accumulation of damaged mitochondria. Damaged mitochondria could release damage-associated molecular patterns (DAMPs) such as mtROS and mitochondrial DNA (mtDNA), which in turn activate the NLRP3 inflammasome and promote IL-1*β* and IL-18 generation [2, 55]. Mitochondria were also the main victims of ROS attack and thus created a vicious cycle [38]. Fortunately, this circle could be then reversed by ROS scavengers [56]. Next, we investigated the scavenge effect of TRIM72 on mtROS and damaged mitochondria in C2C12 cells. As expected, LV-TRIM72 pretreatment attenuated the intracellular accumulation of mtROS induced by AA, and this effect was reversed by addition of si-TRIM72. However, neither LV-TRIM72 nor si-TRIM72 single treatment exhibited significant clearance capability on mtROS (Figures 4(a) and 4(b)). This might because that mtROS amount is low, and TRIM72 has minimal effect on healthy cells. Elevated mtROS level after the addition of CQ suggested the importance of autophagy in ROS clearance (Figures 4(a) and 4(b)). Furthermore, we also disrupted the membrane integrity of mitochondria by CCCP to assess the mitophagy flux. CCCP+LV-TRIM72 presented an increased mitophagy flux compared to CCCP-treated cells and an opposite result after the addition of si-TRIM72 (Figures 4(c)–4(e)). Above all, our results indicated that TRIM72 mitigated the mtROS severity through a mitophagy-dependent way.

The autophagosome formation implies that the early stage of mitophagy flux was initiated, and the autolysosome formation means that the late stage of the flux was completed [10]. Autophagosome formation is a sequential process mainly including three complexes: ULK1 complex, PI3KC3 complex, and ATG16L1 complex [52]. The induction of autophagy begins with phagophore, a nascent membrane structure of autophagosome [57]. The activation of ULK1 complex is essential for phagophore and regulated by PI3K-AKT-mTOR pathway [39]. Recent studies have shown that TRIM72 could suppress the PI3K-AKT-mTOR pathway induced by hypoxia [58]. Here, we also found that AAV-TRIM72 decreased the p-PI3K and p-AKT expression (Figures 6(a)–6(c)). ATG5, one part of ATG16L1 complex, is indispensable for the phagophore elongation and one of the indicators for autophagosome formation [10, 57]. It has been clearly demonstrated that knockout of mitophagy key genes, such as ATG5, results in increased ROS levels in animal models [2, 59]. In the present study, the lack of ATG5 indicated impaired early stage of mitophagy flux in mdx mice, while addition of AAV-TRIM72 remedied this deficiency (Figures 6(d)–6(g)). However, whether it was a direct consequence or a concomitant phenomenon caused by TRIM72 requires more evidence. LAMP1, a represented lysosomal functional protein, was decreased in mdx mice, while the TRIM72 overexpression rise its amount (Figures 5(j)–5(m)). Additionally, the TRIM72 overexpression alleviated the autophagy suppressed by CQ, the effect of which was reversed by si-TRIM72 (Figures 5(a)–5(i)). Together, our data implied the positive aspect of TRIM72 in autophagy both in early and late stages.

Although we support the protective role of TRIM72 in mdx mice, there are also some limitations that could not be ignored. Firstly speaking, the evidence is limited due to the lack of TRIM72 knockout in vivo, despite si-TRIM72 was applied to suppress the effect of LV-TRIM72 in C2C12 cells. Secondly, intravenous injection of AAV9 might reach multiple muscles and have better efficiency than intramuscular injection in widespread muscle disorders such as DMD. And we are preparing experiment to compare the effects of two injection methods. Thirdly, there are obstacles moving AAV-mediated gene therapy strategies to clinical use for little is known about possible serious adverse events [60]. Fourthly, whether NLRP3 inflammasome blockade could reverse the mitophagy in mdx mice would be a charming direction worth exploring. Further experiments would be required to breakdown the limitations mentioned above in future.

**Figure 7:**
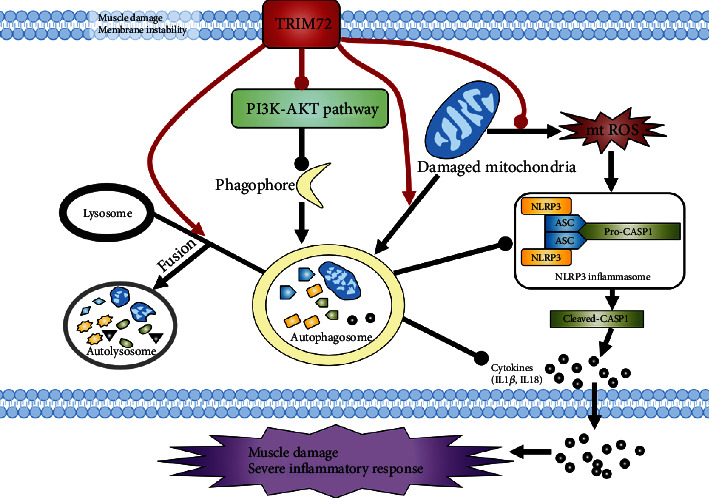
Schematic representation of TRIM72 regulating mitophagy, mtROS, and NLRP3 inflammasome in mdx mice. Muscle damage and membrane instability of mdx mice result in damaged mitochondria, which produce endogenous activator of NLRP3 inflammasome such as mtROS. On the one hand, TRIM72 could scavenge mtROS through mitophagy induction. On the other hand, TRIM72 might facilitate autophagy both in the early and late stages, an indispensable process in removing inflammasome activators (mtROS), inflammasome components, and cytokines. Red lines represent the role of TRIM72. Sharp arrow and round head represent the positive and negative effects, respectively.

Collectively, we demonstrated that the TRIM72 could ameliorate inflammatory response of mdx mice by promoting autophagy-eliminated and suppressing mtROS-activated NLRP3 inflammasome. In addition, TRIM72 might regulate mitophagy both in the early stage by suppressing PI3K-AKT pathway and the late stage by promoting phagolysosome fusion (Figure 7). In the future, TRIM72 might be a promising protein for patients suffering DMD.

## Data Availability

The raw data used to support the conclusions of this study will be available from the corresponding author upon request.

## Supplementary Materials

Supplementary Materials Supplemental Figure 1: TRIM72 was poorly expressed in skeletal muscle of mdx mice. (a) Representative immunofluorescence images of TRIM72 in tibial anterior muscle of WT and mdx mice. TRIM72 (red) and DAPI (blue). Scale bar, 100 *μ*m. (b, c) Western blot analysis and quantification of TRIM72 in skeletal muscles, *n* = 6. Data were expressed as mean ± SEM. ^∗∗^*p* < 0.01. WT: wild type. Supplemental Figure 2: NLRP3 inflammasome was activated, and mitophagy was impaired in skeletal muscle of mdx mice. (a) Representative HE staining images of tibial anterior muscles in WT and mdx mice. Scale bar, 50 *μ*m. (b) Immunofluorescence was performed with specific antibody targeting NLRP3. As shown by arrows in the magnification inset, NLRP3 dots were detected inside myofibers of mdx mice. Scale bar, 50 *μ*m. (c, d) Immunoblot analysis and quantification of NLRP3, ASC, caspase-1, IL18, and IL-1*β* in WT compared with mdx mice, *n* = 6. (e) Serum IL-1*β* level detected by ELISA assay in the two groups, *n* = 6. (f, g) Immunoblot analysis and quantification of P62, LC3, BNIP3L, PARKIN, and TOMM20 in lysates of the two groups, *n* = 6. Data were expressed as mean ± SEM. ^∗^*p* < 0.05, ^∗∗^*p* < 0.01, ^∗∗∗^*p* < 0.001. Supplemental Table 1: antibodies used in the present study.
