# Supplementary material for: TRIM72 Alleviates Muscle Inflammation in mdx Mice via Promoting Mitophagy-Mediated NLRP3 Inflammasome Inactivation
Source: Oxid Med Cell Longev. 2023 Jan 18;2023:8408574. doi: 10.1155/2023/8408574 (PMC9876702; doi:10.1155/2023/8408574)
Supplement: Supplementary Materials — Supplemental Figure 1: TRIM72 was poorly expressed in skeletal muscle of mdx mice. (a) Representative immunofluorescence images of TRIM72 in tibial anterior muscle of WT and mdx mice. TRIM72 (red) and DAPI (blue). Scale bar, 100 μm. (b, c) Western blot analysis and quantification of TRIM72 in skeletal muscles, n = 6. Data were expressed as mean ± SEM. ∗∗p < 0.01. WT: wild type. Supplemental Figure 2: NLRP3 inflammasome was activated, and mitophagy was impaired in skeletal muscle of mdx mice. (a) Representative HE staining images of tibial anterior muscles in WT and mdx mice. Scale bar, 50 μm. (b) Immunofluorescence was performed with specific antibody targeting NLRP3. As shown by arrows in the magnification inset, NLRP3 dots were detected inside myofibers of mdx mice. Scale bar, 50 μm. (c, d) Immunoblot analysis and quantification of NLRP3, ASC, caspase-1, IL18, and IL-1β in WT compared with mdx mice, n = 6. (e) Serum IL-1β level detected by ELISA assay in the two groups, n = 6. (f, g) Immunoblot analysis and quantification of P62, LC3, BNIP3L, PARKIN, and TOMM20 in lysates of the two groups, n = 6. Data were expressed as mean ± SEM. ∗p < 0.05, ∗∗p < 0.01, ∗∗∗p < 0.001. Supplemental Table 1: antibodies used in the present study. [file 8408574.f1.doc]

Supplemental Figure 1.


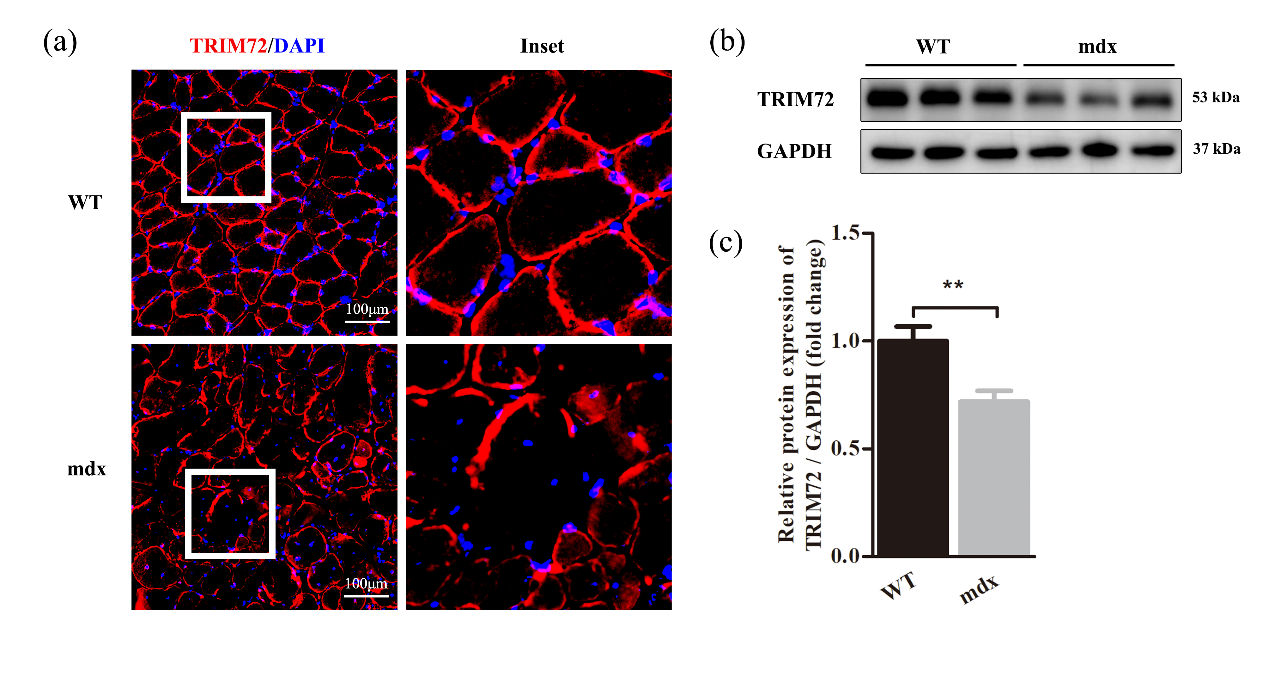


Supplemental Figure 1: TRIM72 was poorly expressed in skeletal muscle of mdx mice. (a) Representative immunofluorescence images of TRIM72 in tibial anterior muscle of WT and mdx mice. TRIM72 (red), DAPI (blue). Scale bar, 100 µm. (b, c) Western blot analysis and quantification of TRIM72 in skeletal muscles. n=6. Data were expressed as mean ± SEM. **p<0.01. WT: wild type.

Supplemental Figure 2.


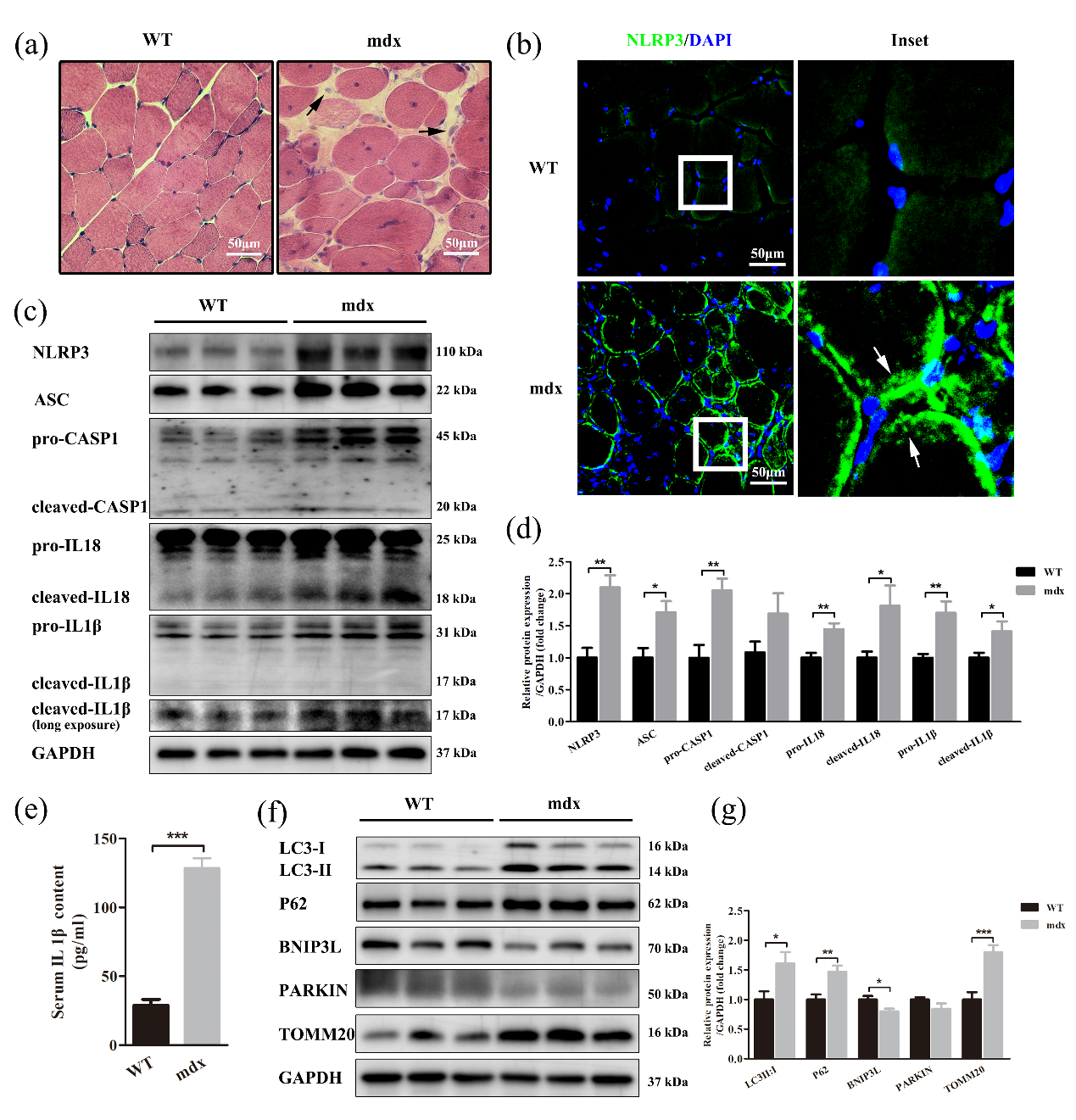


Supplemental Figure 2: [NLRP3](https://www.sciencedirect.com/topics/medicine-and-dentistry/nucleotide-binding-oligomerization-domain-like-receptor) [inflammasome](https://www.sciencedirect.com/topics/medicine-and-dentistry/inflammasome) was activated and mitophagy was impaired in skeletal muscle of mdx mice. (a) Representative HE staining images of tibial anterior muscles in WT and mdx mice. Scale bar, 50 µm. (b)Immunofluorescence was performed with specific antibody targeting NLRP3. As shown by *arrows* in the magnification *inset*, NLRP3 dots were detected inside myofibers of mdx mice. Scale bar, 50 µm. (c, d) Immunoblot analysis and quantification of NLRP3, ASC, Caspase-1, IL18 and IL-1β in WT compared with mdx mice. n=6. (e) Serum IL-1β level detected by ELISA assay in the two groups. n=6. (f, g) Immunoblot analysis and quantification of P62, LC3, BNIP3L, PARKIN, TOMM20 in lysates of the two groups. n=6. Data were expressed as mean ± SEM. *p<0.05. **p<0.01. ***p<0.001.

Supplemental Table 1. Antibodies used in the present study.

| Antibody | | Dilution | Application | Source | Catalog |
| --- | --- | --- | --- | --- | --- |
| Primary antibody | TRIM72 | 1: 5000 | WB | Proteintech | 22151-1-AP |
|  |  | 1: 250 | IF |  |  |
|  | LC3 | 1: 2500 | WB | Proteintech | 14600-1-AP |
|  |  | 1: 250 | IF |  |  |
|  | [P62](https://www.affbiotech.com/goods-4691-AF5384-SQSTM1_p62_Antibody.html) | 1: 1000 | WB | Affinity | [AF5384](https://www.affbiotech.com/goods-4691-AF5384-SQSTM1_p62_Antibody.html) |
|  | ATG5 | 1: 2500 | WB | Proteintech | 10181-2-AP |
|  | LAMP1 | 1: 1000 | WB | Abcam | Ab208943 |
|  |  | 1: 100 | IF |  |  |
|  | BNIP3L | 1: 1000 | WB | Proteintech | 12986-1-AP |
|  | TOM20 | 1: 8000 | WB | Proteintech | 11802-1-AP |
|  | PARKIN | 1: 2000 | WB | Proteintech | 14060-1-AP |
|  | NLRP3 | 1: 1000 | WB | Affinity | DF7438 |
|  |  | 1: 100 | IF |  |  |
|  | TMS1/ASC | 1: 1000 | WB | Affinity | DF6304 |
|  | IL-18 | 1: 8000 | WB | Proteintech | 10663-1-AP |
|  | CASPASE1 | 1: 1000 | WB | Proteintech | 22915-1-AP |
|  | IL1β | 1: 1000 | WB | Abcam | ab234437 |
|  | AKT | 1: 1000 | WB | Cell signaling technology | 4691 |
|  | p-AKT | 1: 1000 | WB | Cell signaling technology | 4060 |
|  | PI3K | 1: 1000 | WB | Affinity | AF6241 |
|  | p-PI3K | 1: 1000 | WB | Affinity | AF3241 |
|  | GAPDH | 1: 1000 | WB | Cell signaling technology | 5174 |
| Secondary antibody | HRP-linked | 1: 2000 | WB | Cell signaling technology | 7074 |
|  | Alexa Fluor 488 | 1: 200 | IF | Abcam | ab150077 |
|  | Alexa Fluor® 594 | 1: 200 | IF | Abcam | ab150088 |
|  | Alexa Fluor® 647 | 1: 200 | IF | Abcam | Ab150115 |
